# Post Mortem Paliperidone Blood Concentrations Following Long-Acting Injectable Treatments

**DOI:** 10.3390/diagnostics15101290

**Published:** 2025-05-21

**Authors:** Pietro Zuccarello, Giulia Carnazza, Antonino Petralia, Nunziata Barbera

**Affiliations:** 1 Department of Psychology and Health Sciences, Pegaso Telematic University, 80143 Naples, Italy; 2 Department of Medical, Surgical and Advanced Technologies Sciences “G.F. Ingrassia”, University of Catania, 95125 Catania, Italy; giulia.carnazza@outlook.it (G.C.); nbarbera@unict.it (N.B.); 3 Psychiatry Unit, Department of Clinical and Experimental Medicine, School of Medicine, University of Catania, 95123 Catania, Italy; petralia@unict.it

**Keywords:** paliperidone, whole blood, small sample volume, LC-MS method, antipsychotics, LAI formulation, post-mortem, autopsy

## Abstract

**Background/Objectives:** I Paliperidone is an antipsychotic recently added into the market in various formulations. There are few data about safety and on therapeutic, toxic, or lethal blood concentrations. Currently, the published analytical methods are often applied to serum or plasma that are not obtained from cadaveric blood. Alternatively, aliquots of high volume of whole blood are used, but often in forensic investigations using samples at very small quantities. The aims of the present study were (a) to develop an analytical method to detect and quantify paliperidone in whole blood using only a small sample volume (10 µL) and (b) to summarize data on post-mortem blood analysis obtained from authentic autopsy cases. **Methods:** Method validation was carried out on 10 µL of whole blood, extracted by LLE and analyzed by LC-MS. Paliperidone concentrations obtained from blood analysis of 16 authentic autopsy cases were reported. **Results:** The method showed a good linearity and sensitivity, a normal distribution, the absence of anomalous values, an interday RSD% always less than 10%, and an 80–120% recovery, as required by AAFS guidelines. Femoral blood concentrations obtained from authentic autopsy cases ranged between 23.4 and 146.9 ng/mL. **Conclusions:** This method is to be used properly in all cases where it is necessary (a) to monitor the therapeutic adherence of patients, (b) to establish the psycho-physical conditions of the treated subject at the time of the death, and (c) to ascertain if the drug may have played a causal role in the obitus. This study reported the first data obtained from post-mortem investigation of subjects treated with paliperidone LAI. Cadaveric blood concentrations could be higher than ante-mortem reference values due to post-mortem redistribution.

## 1. Introduction

Paliperidone, also known as 9-OH-risperidone, is a drug belonging to the class of atypical antipsychotics; it is also the major active metabolite of risperidone. This drug is prescribed for the treatment of schizophrenic or schizoaffective disorders, both as monotherapy and in combination with mood stabilizers [1].

Paliperidone was first marketed in 2007 as an extended-release tablet formulation. Since 2014, the sale of paliperidone palmitate in the Long-Acting Injectable (LAI) formulation has been authorized.

Long-Acting Injectable formulations are useful in the treatment of schizophrenic patients with poor medication adherence due to their ability to maintain the therapeutic plasma level steadily without daily administration [2].

In the literature, there are still few data on the pharmacokinetic and safety of LAI paliperidone palmitate. It was reported that a patient exhibited extrapyramidal symptoms for 5 months after a single injection of LAI paliperidone palmitate [3]. During that period, every attempt to ameliorate this condition turned out to be a failure. It was also reported that a 21-year-old male accidentally received 624 mg paliperidone palmitate intramuscularly, with no reported side effects after 2 weeks of monitoring and observation [4].

In recent decades, there was an increasing use of antipsychotics, with a growth in morbidity and mortality due to accidental overdose or for suicidal intention [5]. Antipsychotics are among the substances most frequently detected in post-mortem forensic investigations [6], and overdose is quite common.

More and more common are the forensic investigations aimed at finding possible misconduct of medical doctors or nurses who could have prescribed or administered excessive drug dosages that led to lethal poisoning. It is essential to have information to establish whether a possible overdose may have a causal or concausal role in death. In these cases, the knowledge of normal, toxic, or lethal ante-mortem and post-mortem concentrations is tantamount, although they are not always available [7]. In living subjects, the mean serum concentration range of paliperidone, as reported in previous studies, is 36 ± 25 ng/mL [8], but a high variability of blood concentrations was also observed at the same dosage with LAI formulations [9]. A clinical study reported mean plasma paliperidone values following daily oral administrations of 40.2 ± 19.8 ng/mL (dose: 9 mg per day) and 44.2 ± 15.9 ng/mL (dose: 12 mg per day). In contrast, the same study reported mean plasma paliperidone values following administration of the Long-Acting Injectable formulations (dose: 100 mg per 28 days) of 32.9 ± 12.7 ng/mL and 49.9 ± 25.9 ng/mL (dose: 150 mg per 28 days) [10].

Currently, the analytical methods described in the literature are often applied to serum or plasma, rather than to whole blood. Some of these methods also require long times for extraction with SPE or LLE methods; moreover, some of these methods are not always applicable given the amount of blood required, which cannot always be available at time of autopsy, particularly in cases of bleeding death.

The aims of the present study were to develop an analytical method to detect and quantify paliperidone in whole blood using only a small sample volume (10 µL) and to summarize data obtained from authentic autopsy cases.

## 2. Materials and Methods

### 2.1. Chemicals and Reagents

Reference standards of paliperidone (purity > 98%) were purchased from Sigma Aldrich-Merck (Darmstadt, Germania). Clozapine was obtained by Sandoz (Trento, Italy). Ultrapure water (LC-MS/MS grade), formic acid (LC-MS grade), acetonitrile (LC-MS/MS grade), and sulfuric acid (for analysis-ISO) were purchased from Carlo Erba Reagents (Milan, Italy).

### 2.2. Calibration Standards and Quality Control Samples

The reference standard was obtained by dissolving paliperidone in methanol to obtain the concentration of 100 µg/mL; subsequent dilutions in methanol at suitable concentrations were further carried out. The calibration curves were obtained by spiking blank blood samples with paliperidone at concentrations of 10, 20, 50, 100, and 200 ng/mL.

### 2.3. Sample Extraction and Analysis

The extraction procedure, described in a previous study [5], was applied on aliquots of 10 µL of whole blood and urine. Each sample was added with clozapine (Internal Standard I.S.; 100 ng/mL), 10 µL of 0.1 N sulfuric acid, and 30 µL of acetonitrile. After agitation and ultra-centrifugation, the supernatant was transferred to vials and analyzed by LC-MS/MS under the same conditions previously described [5]. We used a Waters UHPLC-ESI-TQD Acquity system H-class with Acquity UPLC^®^ HSS T3 C18 1.8 µm–2.1 × 100 mm (Milford, MA, USA). Table 1 is a schematized representation of the ion transitions selected for paliperidone and clozapine.

### 2.4. Method Validation

Linearity, interday accuracy, intra- and interday precision, limit of detection (LOD), lower limit of quantification (LLOQ), matrix interferences, and carryover were assessed for all matrices with the same validation protocol as the previous study [5], in accordance with the Academy Standards Board Standard Practices for Method Development in Forensic Toxicology [11,12].

In the present study, linearity was verified on 5-point calibration curves and replicated five times. Intra- and interday (five day) precision and recovery were evaluated at three concentrations of 20, 50, and 100 ng/mL. Ten different sources (dead (*n* = 5) and living (*n* = 5)) were used to assess the matrix effects.

### 2.5. Paliperidone Concentrations in Authentic Autopsy Cases

The samples of 35 autopsies analyzed by the Forensic Toxicology Laboratory of the University of Catania from 2017 to 2024 were included in the study. All patients were treated with paliperidone LAI at different dosages and formulations: 175 mg/quarter (*n* = 1), 263 mg/quarter (*n* = 3), 350 mg/quarter (*n* = 4), 525 mg/quarter (*n* = 3), 100 mg/month (*n* = 1), and 150 mg/month (*n* = 4). The deaths were not related to paliperidone. Femoral blood samples, collected during autopsy, added with 200 mg of sodium fluoride and 30 mg potassium oxalate, and stored at −20 °C, were analyzed applying the described method.

## 3. Results

### 3.1. Method Validation

For whole blood samples, the calibration curve of paliperidone showed a mean linearity coefficient r^2^ equal to 0.9992. The LOD was 5 ng/mL while the LLOQ was 15 ng/mL. Table 2 summarizes the calibration range and related correlation coefficient (r^2^), LOD, and LLOQ.

The method showed a normal distribution, the absence of anomalous values, an interday RSD% of 9.2% and an interday recovery of 112.0% at the concentration of 20 ng/mL, an interday RSD% of 6.0% and an interday recovery of 95.6% at the concentration of 50 ng/mL, and an interday RSD% of 6.5% and an interday recovery of 96.8% at the concentration of 100 ng/mL.

Table 3 schematizes the results concerning the method performance at each concentration level. Mean value, deviation standard, relative deviation standard RSD%, and percental recovery R% are calculated.

No matrix interference or internal standard interference was observed. No analytical interference was shown for the presence of common antipsychotics or antidepressants. The matrix does not significantly reduce the peak intensity of paliperidone (<10%). No carryover has been observed.

### 3.2. Paliperidone Concentrations in Authentic Autopsy Cases

Femoral blood paliperidone concentrations (ng/mL) obtained from 16 authentic autopsy cases are summarized in Table 4.

In the subject treated with paliperidone LAI 175 mg per quarter, the blood concentration was 31.9 ng/mL.

In the three subjects treated with paliperidone 263 mg per quarter, the mean blood concentration was 48.0 ng/mL [ranged from 24.9 to 62.1 ng/mL].

In the four subjects treated with paliperidone 350 mg per quarter, the mean blood concentration was 48.3 ng/mL [ranged from 30.6 to 99.8 ng/mL].

In the three subjects treated with paliperidone 525 mg per quarter, the mean blood concentration was 84.2 ng/mL [ranged from 45.2 to 146.9 ng/mL].

In the subject treated with paliperidone 100 mg per month, the blood concentration was 48.6 ng/mL.

In the four subjects treated with paliperidone 150 mg per month, the mean blood concentration was 73.8 ng/mL [ranged from 23.4 to 142.6 ng/mL].

In the totality of the cases, paliperidone concentrations ranged between 23.4 and 146.9 ng/mL.

## 4. Discussion

Since the mid-1950s, neuroleptic drugs have been considered the primary treatment for both acute and chronic schizophrenia. Neuroleptic drugs, also called antipsychotics, effectively relieve the signs and symptoms of acute schizophrenia, preventing relapses in stabilized patients [13].

These drugs are divided into two groups: (a) typical antipsychotics, also called conventional antipsychotics, first-generation antipsychotics, or major tranquilizers; (b) atypical antipsychotics, also known as second generation antipsychotics [14].

Antipsychotics are often taken excessively—accidentally or voluntarily—increasing the risk of fatal overdose, as frequently recorded in poison control centers. In fact, in 2009, about 43,000 cases of overdose were reported at the “U.S Poison Centers”; in 2010, more than 4000 calls were recorded to the “California Poison Control System (CPCS)” regarding excessive exposure to drugs (most of which were atypical antipsychotics) among both pediatric and adult subjects; moreover, most of these cases were due to voluntary ingestion [15].

The ranges of therapeutic concentrations of some antipsychotics are not always available as newly authorized drugs. However, in post-mortem investigations, having reference concentration ranges is essential to diagnose poisoning.

There are not yet sufficient data in the literature about post-mortem paliperidone blood concentrations, especially following LAI administration. Most of the available data concern 9-OH-Risperidone, produced by metabolism after risperidone administration. However, paliperidone is also a relatively recent drug. It is available in different formulations: oral and long-acting intramuscular injections.

For forensic purposes, a simple and rapid analytical method for its determination in whole blood has not been published yet.

The present method, applied in whole blood samples, resulted in effective and efficient qualitative and quantitative determination of paliperidone, using extremely small quantities of sample (10 microliters). The method has also proven to be rapid and economic. The calibration curve of paliperidone showed a mean linearity coefficient r^2^ equal to 0.9992. LOD was 5.0 ng/mL while LLOQ was 15.0 ng/mL. The method showed a normal distribution, the absence of anomalous values, an interday RSD% always less than 10%, and an 80–120% recovery, as required by AAFS guidelines.

Other validated methods for paliperidone determination are reported in the literature.

De Meulder et al. validated an LC-MS/MS method for paliperidone detection in serum using solid-phase extraction (SPE) with a 200 μL sample volume [16]. Fisher et al. developed a similar approach, employing liquid–liquid extraction with a butyl acetatebutanol mixture, followed by centrifugation [17]. Proença et al. described a method for whole blood analysis in post-mortem cases, utilizing SPE and elution with methanol, followed by evaporation and reconstitution in an acetonitrile–formic acid mobile phase [18].

In the forensic field, the main limitation of the methods described by De Meulder et al. and by Fisher et al. is the use of serum rather than whole blood. Since it is impossible to obtain serum from cadaveric blood, for forensic investigations, it is necessary to use a method validated on whole blood. The method reported by Proença et al., even if validated on whole blood, is extremely time-consuming; furthermore, the main limitation of this method is the considerable volume of sample required (500 µL). In forensic investigations, it could be necessary to have methods capable of using the smallest possible amount of samples, for example, when blood sample is not abundant due to corpse decomposition or in cases of bleeding deaths, and the sample taken must be sufficient to carry out all the analyses that the case requires.

The present method, applicable to 10 µL aliquots of whole blood, could be useful in investigations aimed at establishing the psycho-physical conditions of the treated subject at the time of the death, as well as to ascertain if the drug may have played a causal role in the exitus. The possibility of analyzing such small volumes of whole blood could facilitate investigations in cases where it is not possible to collect adequate quantities during the autopsy, such as in cases of deaths that occurred from abundant blood loss or in cases where the onset of putrefactive processes reduces the quantity of all biological fluids, which must also be sufficient to analyze other substances.

The method could also be used in clinical cases to monitor therapeutic compliance and to obtain information about the dose-related adverse effects at toxic or lethal concentrations.

Regarding paliperidone values, all authentic post-mortem cases showed paliperidone concentrations between 23.4 and 146.9 ng/mL.

Data were also divided based on the posology of paliperidone formulation. In the subject treated with paliperidone LAI 175 mg/quarter, the blood concentration was 31.9 ng/mL. In the three subjects treated with paliperidone 263 mg/quarter, the mean blood concentration was 48.0 [range: 24.9–62.1] ng/mL. In the four subjects treated with paliperidone 350 mg/quarter, the mean blood concentration was 48.3 [range: 30.6–99.8] ng/mL. In the three subjects treated with paliperidone 525 mg/quarter, the mean blood concentration was 84.2 [range: 45.2–146.9] ng/mL. In the subject treated with paliperidone 100 mg/month, the blood concentration was 48.6 ng/mL. In the four subjects treated with paliperidone 150 mg/month, the mean blood concentration was 73.8 [range: 23.4–142.6] ng/mL.

Helland and Spigset carried out a clinical study on subjects treated with paliperidone LAI. The mean serum concentration of paliperidone was approximately 13 ng/mL after an intramuscular dose of 50 mg/month, approximately 23 ng/mL after a dose of 75 mg/month, 25 ng/mL after a dose of 100 mg/month, and 40 ng/mL after a dose of 150 mg/month. The maximum concentration obtained in all cases was approximately 51 ng/mL [9].

Our data, obtained from cadaveric femoral blood samples, generally showed concentrations higher than those reported in clinical studies. Although the mean values are not very different from those found in living subjects, in some cases, the blood concentration was more than double the maximum ante-mortem reference values. As is well known, blood concentrations may increase due to post-mortem redistribution [19]. A study on the post-mortem redistribution of paliperidone, as risperidone metabolite, reports central/femoral blood ratios between 1.3 and 2.0. This confirms that paliperidone undergoes modest redistribution [20]. Another study, on the other hand, showed a reduction in post-mortem blood concentration, indicating poor stability of the analyte in cadaveric blood [21]. However, a further study on the paliperidone metabolite investigated the stability of the substance in whole blood samples spiked with 200 mg of sodium fluoride and 30 mg of potassium oxalate and stored at 4 °C, −20 °C, and −60 °C; the study showed the stability of the substance under these conditions [22]. Another study investigated the stability of paliperidone in blood samples stored at 20 °C, 4 °C, −20 °C, and −60 °C. It was observed that when the blood samples were stored at 20 °C, there was a minimal loss between 15 and 30%. The stability was demonstrated when blood samples were stored at 4 °C, −20 °C, and −60 °C [23].

Therefore, in order to ensure the stability of paliperidone, in the present study, each blood sample, collected during autopsy, was spiked as suggested with 200 mg of sodium fluoride and 30 mg of potassium oxalate and stored at −20 °C.

An important aspect to consider when evaluating increased post-mortem concentrations in cases of LAI formulation treatment is the possibility of the drug, administered into the muscle, continuing to be released into the blood after death. This diffusion mechanism is currently studied only in vivo. In fact, in a study conducted on olanzapine pamoate administered in dogs through the intramuscular route, an increase in concentration in the post-mortem of approximately 7 times compared to the values determined in the ante-mortem was observed. The authors concluded that this phenomenon seems to be attributable to intramuscular administration [24]. Therefore, although this has not yet been ascertained in human cadavers, it is advisable for the toxicologist to be well informed on the formulation of paliperidone administered before death (oral or LAI) to also consider this phenomenon in the interpretation of the data, especially if the blood value is above the normal range.

Furthermore, post-mortem tissue acidification, especially blood, may promote ester hydrolysis and increase unbound paliperidone in the blood.

However, the mean values reported in the present study on cadaveric blood are not very far from those obtained from living subjects in another clinical study [10]. In the subject treated with paliperidone 100 mg/month before death, the cadaveric blood concentration was 48.6 ng/mL, whereas in the clinical study, the mean value was 32.9 ± 12.7 ng/mL [ranging from 20.2 to 45.6 ng/mL] for a dose of 100 mg per 28 days. In the four subjects treated with paliperidone 150 mg/month before death, the mean cadaveric blood concentration was 73.8 ng/mL, whereas in the clinical study, the mean value was 49.9 ± 25.9 ng/mL [ranging from 24.0 to 75.8 ng/mL] for a dose of 150 mg per 28 days. In both cases, the mean cadaveric blood concentration was next to the upper limit of therapeutic range for the respective dose.

In summary, data obtained in the present study could provide valuable information for the interpretation of positive data in post-mortem investigations. According to the present data, it could be hypothesized that post-mortem blood concentrations of paliperidone up to 150 ng/mL should be considered to be associated with a therapeutic dosage of the drug.

Some limitations of the study can be highlighted:

1. It would have been useful to analyze also the central cadaveric blood in order to estimate the potential of redistribution;
2. It was not possible to correlate the results with the post-mortem interval;
3. It was not possible to correlate the results with the interval since the last administration;
4. A preliminary study on the stability of paliperidone was not performed on the investigated samples.

## 5. Conclusions

### 5.1. Key Findings

The present validated method carried out on a 10 µL cadaveric blood sample could be useful for the determination of paliperidone in forensic investigations, thanks to use of an extremely small sample volume and the quick execution of the analysis.

The application of this method on real cadaveric samples provided data about therapeutic concentrations found post-mortem.

### 5.2. Forensic Applications

Data obtained in the present study on authentic autopsy cases provide valuable information for the interpretation of a positive data in post-mortem investigations, where the subject was treated with paliperidone by Long-Acting Injectable formulation before death.

### 5.3. Future Research Directions

Cadaveric blood concentrations could be higher than ante-mortem reference values probably due to the post-mortem redistribution, and possibly due to the continued release of the drug from the muscle to the hematic torrent. Future studies should aim to clarify the impact of LAI formulations on post-mortem redistribution.

## Figures and Tables

**Table 1:** Paliperidone and related ion transitions (*m*/*z*).

Analytes	Precursor (*m*/*z*)	Product (*m*/*z*)	Cone (V)	Collision (V)
**Paliperidone**	427.0	206.5	40	45
	427.0	109.9	40	50
**Clozapine**	327.2	269.9	35	20

**Table 2:** Calibration ranges and related curve correlation coefficients r^2^, LOD, and LLOQ.

Calibration Range	Units	r^2^ (*n* = 5)	LOD	LLOQ
**10** **–** **200**	ng/mL	0.9992	5	15

**Table 3:** Method performance parameters.

	Units	Target Value	Interday Mean (n = 15)	Interday RSD% (n = 15)	Interday R% (n = 15)	Matrix Effect % (n = 10)	CV% (n = 10)
**Blood**	ng/mL	20	22.4	9.2	112.0	−7.1	9.2
		50	47.8	6.0	95.6	−5.9	7.6
		100	96.8	6.5	96.8	−5.5	8.1

**Table 4:** Paliperidone concentrations (ng/mL) in whole blood of post-mortem cases.

	*n*	Mean	Minimum	Maximum
**175 mg/quarter**	1	31.9	/	/
**263 mg/quarter**	3	48.0	24.9	62.1
**350 mg/quarter**	4	48.3	30.6	99.8
**525 mg/quarter**	3	84.2	45.2	146.9
**100 mg/month**	1	48.6	/	/
**150 mg/month**	4	73.8	23.4	142.6

## Data Availability

All data of the present study are available at Forensic Toxicology Laboratory of University of Catania.
